# Knowledge and awareness of hepatitis B among households in Malaysia: a community-based cross-sectional survey

**DOI:** 10.1186/s12889-018-6375-8

**Published:** 2019-01-10

**Authors:** Yogambigai Rajamoorthy, Niazlin Mohd Taib, Subramaniam Munusamy, Samsul Anwar, Abram Luther Wagner, Mudatsir Mudatsir, Ruth Müller, Ulrich Kuch, David Alexander Groneberg, Harapan Harapan, Aye Aye Khin

**Affiliations:** 1 Department of Economics, Faculty of Accountancy and Management, Universiti Tunku Abdul Rahman, Sungai Long Campus, Jalan Sungai Long, Cheras, 43000 Kajang, Selangor Malaysia; 2 Department of Medical Microbiology and Parasitology, Faculty of Medicine and Health Science, Universiti Putra Malaysia, UPM Serdang, Selangor, Malaysia; 3 School of Management and Business, Manipal International University, Putra Nilai, Negeri Sembilan Malaysia; 4 Department of Statistics, Faculty of Mathematics and Natural Sciences, Universitas Syiah Kuala, Banda Aceh, Indonesia; 5 Department of Epidemiology, University of Michigan, Ann Arbor, MI USA; 6 Department of Microbiology, School of Medicine, Universitas Syiah Kuala, Banda Aceh, Indonesia; 7 Medical Research Unit, School of Medicine, Universitas Syiah Kuala, Jl. T. Tanoeh Abe, Darussalam, Banda Aceh, 23111 Indonesia; 8 Tropical Disease Centre, School of Medicine, Universitas Syiah Kuala, Banda Aceh, Indonesia; 9 Institute of Occupational Medicine, Social Medicine and Environmental Medicine, Goethe University, Frankfurt am Main, Germany; 10 Unit of Medical Entomology, Institute of Tropical Medicine, Antwerp, Belgium; 11 School of Biomedical Sciences, University of Western Australia, Nedlands, Western Australia Australia

**Keywords:** Hepatitis B, Knowledge, Awareness, Hepatitis B vaccination, Hepatitis B infection

## Abstract

**Background:**

Hepatitis B (HepB) is a major public health concern in Malaysia yet little is known about knowledge and awareness of this infection in the country. Such information is essential for designing effective intervention strategies for HepB prevention and control. The aim of this study was to characterize knowledge and awareness regarding HepB in Malaysia and to identify their associated sociodemographic determinants.

**Methods:**

A community-based cross-sectional survey was conducted between January and May 2016 in Selangor state of Malaysia. A two-stage cluster random sampling design was used and one adult member of selected households was interviewed face-to-face. Logistic regression was used to estimate the differences in knowledge and awareness between groups.

**Results:**

A total of 764 households completed the interviews and were included in the final analysis. Only 36.9 and 38.8% of the participants had good knowledge and awareness, respectively. The factors associated with good knowledge were being in the 35–44 year age group, Malay ethnicity, high educational attainment and high family income. Being Chinese, being older and having high educational attainment were determinants of having good awareness towards HepB. Participants who had good knowledge were 2.5 times more likely to also have good awareness (OR: 2.41, 95% CI: 1.78–3.26, *p* < 0.001).

**Conclusions:**

This study reveals a low level of knowledge and awareness of HepB among households in Malaysia. This finding highlights the need to improve public knowledge and awareness through well-designed programs targeting vulnerable groups in order to reduce hepatitis B virus transmission and achieve the governmental target of eliminating viral hepatitis as a public health concern by 2030.

**Electronic supplementary material:**

The online version of this article (10.1186/s12889-018-6375-8) contains supplementary material, which is available to authorized users.

## Background

An estimated 257 million people were living with hepatitis B virus (HBV) infection globally in 2015 [1]. Approximately 887,000 deaths were associated with the two main hepatitis B (HepB) complications: cirrhosis and hepatocellular carcinoma [1]. Globally, the prevalence of HepB surface antigen (HBsAg) was 3.61% and is highest in the African region [2]. In Southeast Asia, an estimated 2.0% of the general population are infected [2]. In Malaysia, whose population was over 31 million in 2016, an estimated 1 million people are chronically infected with HBV [3], and this infection continues to be a major health problem in the country [3, 4]. In 2014, data from the Malaysian Ministry of Health revealed that the number of deaths due to HepB is greater than any other vaccine-preventable disease in Malaysia [5].

In 2015, the World Health Organization issued the Glasgow Declaration on Hepatitis Elimination. The declaration outlines the commitments necessary to eliminate viral hepatitis as a public health concern by 2030 [6]. To achieve this target, the Malaysian Ministry of Health and related, multi-sectoral organizations are working together to develop a strategic road map to eliminate viral hepatitis in Malaysia by 2030 [3]. One of the most important components in the strategic road map adopted by Malaysia is the widespread use of preventive measures such as vaccination. These preventive measures should not only be conducted by the government but should also include participation and buy-in from community members. Therefore, information related to knowledge and awareness of community members is crucial in order to design prevention programs in the community. However, information about HepB knowledge and awareness is limited in Malaysia. Previous studies of knowledge have focused only on individuals with chronic hepatitis B attending a hepatology clinic [7], on university students [8] and on community members in specific locations, such as Puchong [9] or Kuala Lumpur and Selangor [10]. Two previous studies have investigated awareness of HepB in Malaysia [10, 11].

The scarcity of available scientific literature indicates the need for more comprehensive data related to knowledge and awareness among Malaysians in order to inform national programs and policies. The aim of this study was to assess knowledge and awareness of HepB and to identify associated sociodemographic determinants among representative community members in Malaysia. Findings from this study can be used to design intervention strategies at a national scale and to develop an effective HepB prevention program.

## Methods

### Study setting

Between January and May 2016, a cross-sectional survey was conducted in Selangor state which is located on the west coast of peninsular Malaysia and which encircles the capital Kuala Lumpur. The state is the most populous state in Malaysia with a surface area of 8104 km^2^ and a population of 5.79 million. The design, setting, analyses and reporting of this study adhered to the STROBE guidelines for cross-sectional studies in epidemiology [12]. See Additional file 1 for the detailed checklist of STROBE criteria.

### Sampling method

Based on a minimum recommended sample size calculation, along with Selangor’s population (*5.79 million people)* [13], this study required 385 respondents. This sample size is based on the conservative assumption that 50% of participants would have good knowledge and awareness of HepB, with a 5% margin of error and a confidence interval of 95%. Because this study was part of a research project in which the main objective was to assess the willingness-to-pay (WTP) for HepB vaccine [14], the sample size was also calculated to answer that project’s objective. Based on the assumption that the deviation of the estimated WTP from the true value (∆) was 15%, the relative error of the true WTP (V) was 2.0 with a 5% margin of error and a confidence interval of 95%, the minimum sample size required for the project was 683. This sample size was acceptable to assess the knowledge and attitude of HepB in this article.

To capture a representative sample from the population, a two-stage cluster sampling design with proportional allocation was employed with assistance from the Malaysia Department of Statistics. First, Selangor state was divided into 16,562 small enumeration blocks with each enumeration block consisting of 80–120 living quarters. Sixty four out of 16,562 enumeration blocks were selected randomly. Second, 12 out of 80–120 living quarters were randomly selected within each enumeration block. This sampling scheme resulted in a total of 768 living quarters, at which location one adult who was aged 20 years or older and who was a Malaysian citizen was selected and invited to participate.

### Data collection

Prior to enrolment, the selected household members were informed of the study aims. Once the participants agreed to participate, a face-to-face interview was conducted in Malay or English by trained interviewers. Participants were also informed that they could quit at any time during the interview session. In case the participant was unable to understand either languages, the interview was conducted with a translator who was able to communicate and translate the questionnaire into the preferred language.

### Questionnaire development and testing

A set of structured questionnaires, used to guide the interview, was developed based on the existing literature and then translated to the Malay language. The questionnaire covered knowledge and awareness of HepB and demographic data. See Additional file 2 for the detailed questionnaire. In this study, knowledge and awareness were differentiated such that knowledge assessed detailed and factual information about HepB, whereas awareness was associated with information that is personally relevant [15]. The knowledge domain included questions on the causative agent, transmission, preventive measures, signs and symptoms, and treatment for HepB. In contrast, the awareness domain assessed whether respondents were aware of their own or their family members’ HepB status and HepB vaccination status. Prior to its use in the actual study, the questionnaire was tested in a pilot study which consisted of 121 respondents selected from a public place in the Serdang area. The reliability of the questionnaire was considered to have moderate internal consistency and had sufficient to acceptable values for research purposes because the overall Cronbach’s alpha was 0.6.

### Study variables

#### Response variables

The response variables in this study were knowledge and awareness of HepB among community members in Malaysia. A total of 22 questions were used to measure knowledge of HepB. Possible responses to all of the questions were “yes” or “no”; there was no “do not know” option. A correct response for knowledge questions was given a score of one, whereas an incorrect response was given a score of zero. For each participant, the knowledge score was calculated as the total sum of correct responses and therefore higher scores indicate better knowledge. The awareness domain was measured using a set of four questions. For this domain, a score of one was given if respondents indicate “yes”, showing that they were aware of HepB, whereas “no” and “do not know” responses were given a score of zero, indicating that respondents were not aware of HepB. Hence, higher scores indicated better awareness of the disease.

#### Explanatory variables

Through an intensive literature review [9–11, 16–19], the following socioeconomic variables were included in this study: gender, age, ethnicity, marital status, employment status, educational attainment and family income level. The ethnicity of the participants was categorized as Malay, Chinese, Indian and others. Educational attainment, defined as the highest level of formal education completed, was classified into illiterate or primary school, secondary school, diploma, degree and postgraduate. Participants’ age was divided into four groups (25–34, 35–44, 45–54 and 55 years old or above). For employment status, five general types were used for classification: public sector, private sector, self-employment, student or university student and retired. Participants who had an unclassified job were grouped as others, and participants who had no job currently were listed as unemployed. Family income was assessed by asking the participants to calculate the average amount of money earned each month.

### Statistical analysis

The statistical approaches in this study have been published elsewhere [20–24]. Within the knowledge and awareness domains, correct responses were summed for a total possible score of 22 and 4 points, respectively. The differences in the mean score of knowledge and awareness between explanatory variables were analyzed using an Analysis of Variance (ANOVA). In addition, the knowledge and awareness domains were dichotomized into “good” and “poor” based on a 75% cut-off point (i.e. ≥17 points for good knowledge, and ≥ 3 points for good awareness). Previous literature has used a similar cut-off point of either 80% (for example, in studies in Indonesia [24–28] and Nepal [29]), or 75% [21, 30].

To assess the association between the explanatory variables and the response variables, a multi-step logistic regression analysis was employed. First, all explanatory variables (gender, age, ethnicity, marital status, employment status education level and family income level) were included in separate univariate logistic regression analyses. In the next step, explanatory variables that had a *p* ≤ 0.25 in the univariate analysis were entered into the multivariate analysis. Confounding factors were explored as described previously [31]. A Chi-squared analysis was used to assess the association between good knowledge and good awareness. The correlation between scores of knowledge and awareness was assessed using Spearman’s rank correlation (r_s_) based on the Kolmogorov–Smirnov normality test. The 95% confidence intervals (95% CI) for r_s_ were calculated as described previously [32].

## Results

### Socio demographic characteristics

In this study, 768 respondents were interviewed and four responses were excluded due to incomplete information leaving a dataset with a total of 764 (99.4%) participants. This study was a part of a Malaysian Hepatitis B Project and the characteristics of the respondents have been described elsewhere [33]. Briefly, a majority of respondents were male (54.5%) and 78.7% were married. Approximately 2% of the respondents never had formal education and the majority had secondary education (47.3%). More than a third of the respondents were aged between 25 and 34 and the majority of them were Malay (59.4%), followed by Chinese (23.7%) and Indian (16.4%).

### Knowledge of hepatitis B and associated sociodemographic determinants

The mean score of knowledge was 14.94 (±3.75), and there was a statistically significant difference across age, ethnicity, employment status, educational attainment, and family income (Table 1). Out of the total participants, 282 (36.9%) had a good knowledge level. In the univariate analysis, age, ethnicity, educational level and family income were associated with good knowledge. Gender, marital status, and type of employment had no association with participants’ knowledge (Table 1). Compared to the youngest age group (25–34 years), participants aged 35–44 years had 1.54 times higher odds of having good knowledge (OR: 1.54; 95% CI: 1.08–2.20). Malays had 1.85 times higher odds of having good knowledge compared to Indians (OR: 1.85; 95% CI: 1.19–2.86). Compared to individuals who had only completed primary school or who were illiterate, there were increased odds observed among participants who had completed a diploma certificate (OR: 2.72), a bachelor degree (OR: 3.47) or a postgraduate degree (OR: 4.73). A higher monthly income was also associated with good knowledge.

**Table 1:** Characteristics of participants and sociodemographic determinants associated with knowledge towards hepatitis B (*n* = 764)

Variable	Frequency (%)	Mean score (±SD)	*p*-value^a^	Knowledge (good/poor)	Univariate logistic (good vs. poor)		Multivariate logistic (good vs. poor)	
					OR (95% CI)	*p*-value	OR (95% CI)	*p*-value
Gender			0.497					
Male (R)	416 (54.5)	15.07 (3.77)		165/251	1		1	
Female	348 (45.5)	14.78 (3.74)		117/231	0.77 (0.57–1.04)	0.085	0.77 (0.54–1.10)	0.150
Age (years old)			0.001**					
25–34 (R)	275 (36.0)	14.77 (3.79)		95/180	1		1	
35–44	232 (30.4)	15.64 (3.33)		104/128	1.54 (1.08–2.20)	0.018*	1.68 (1.14–2.46)	0.008**
45–54	162 (21.2)	14.89 (3.98)		59/103	1.09 (0.72–1.63)	0.692	1.22 (0.78–1.90)	0.392
55 and above	95 (12.4)	13.81 (3.94)		24/71	0.64 (0.38–1.08)	0.096	0.74 (0.38–1.42)	0.359
Ethnicity			0.002**					
Indian (R)	125 (16.4)	14.05 (4.03)		34/91	1		1	
Malay	453 (59.3)	15.32 (3.52)		185/268	1.85 (1.19–2.86)	0.006**	1.68 (1.07–2.65)	0.026*
Chinese	182 (23.8)	14.56 (4.01)		61/121	1.35 (0.82–2.22)	0.240	1.17 (0.69–1.98)	0.573
Others	4 (0.5)	16.50 (1.73)		2/2	2.68 (0.36–19.76)	0.334	3.69 (0.48–28.54)	0.211
Marital status			0.247					
Single (R)	147 (19.2)	14.48 (4.12)		49/98	1			
Married	600 (78.5)	15.05 (3.67)		225/375	1.20 (0.82–1.76)	0.348	–	–
Widowed and divorced	17 (2.2)	15.18 (3.19)		8/9	1.78 (0.65–4.89)	0.265		
Employment status			0.041*					
Public sectors (R)	101 (13.2)	16.08 (3.00)		45/56	1		1	
Private sectors	222 (29.1)	14.91 (3.89)		85/137	0.77 (0.48–1.24)	0.288	0.92 (0.56–1.53)	0.752
Self-employment	184 (24.1)	14.63 (3.93)		67/117	0.71 (0.44–1.17)	0.179	0.88 (0.51–1.50)	0.633
Student	27 (3.5)	14.67 (3.50)		9/18	0.62 (0.26–1.52)	0.297	0.67 (0.26–1.72)	0.399
Retired	54 (7.1)	14.17 (4.01)		18/36	0.62 (0.31–1.24)	0.177	1.11 (0.49–2.51)	0.803
Unemployed	156 (20.4)	14.90 (3.62)		51/105	0.60 (0.36–1.01)	0.056	1.00 (0.55–1.81)	0.995
Others	20 (2.6)	15.10 (3.87)		7/13	0.67 (0.25–1.82)	0.432	1.13 (0.39–3.29)	0.828
Educational attainment			< 0.001**					
Illiterate or primary school (R)	51 (6.7)	12.45 (4.87)		10/41	1		1	
Secondary education	361 (47.3)	14.83 (3.43)		120/241	2.04 (0.99–4.22)	0.054	1.60 (0.75–3.45)	0.228
Diploma/certificate	193 (25.3)	15.24 (3.80)		77/116	2.72 (1.29–5.76)	0.009**	1.76 (0.77–4.02)	0.180
Degree holder	131 (17.1)	15.37 (3.89)		60/71	3.47 (1.60–7.50)	0.002**	2.28 (0.95–5.44)	0.064
Postgraduate	28 (3.7)	16.75 (2.27)		15/13	4.73 (1.72–13.05)	0.003**	3.20 (1.06–9.62)	0.038*
Family income (RM)			0.002**					
≤ 2000 (R)	183 (24.0)	14.08 (4.06)		51/132	1		1	
2001–3000	178 (23.3)	14.79 (3.53)		56/122	1.19 (0.76–1.87)	0.455	1.02 (0.63–1.63)	0.949
3001–4000	125 (16.4)	15.31 (2.88)		45/80	1.46 (0.89–2.37)	0.131	1.22 (0.73–2.04)	0.448
4001–5000	88 (11.5)	15.25 (3.64)		37/51	1.88 (1.10–3.20)	0.020*	1.63 (0.92–2.90)	0.093
> 5000	190 (24.9)	15.52 (4.07)		93/97	2.48 (1.61–3.82)	< 0.001**	1.87 (1.15–3.06)	0.012*

*Significance at 0.05

**Significance at 0.01

^a^Analysis using Analysis of Variance (ANOVA)

*CI* confidence interval, *OR* odds ratio, *R* reference group, *RM* Ringgit Malaysia, *SD* standard deviation

After excluding predictor variables with *P* > 0.25 from the analysis, the multivariate model revealed that age, ethnicity, education and family income were significant predictors of good knowledge (Table 1). Having a postgraduate degree was the strongest predictor factor for good knowledge (OR: 3.20; 95% CI: 1.06–9.62) followed by individuals with the highest family income (OR: 1.87; 95% CI: 1.15–3.06). Being Malay and aged 35–44 years old increased the odds of having good knowledge approximately 1.5 times over the referent category.

### Awareness towards hepatitis B and associated sociodemographic determinants

The mean score of awareness was 1.98 (±1.31), and there was a statistically significant difference across age, ethnicity, marital status, and educational attainment (Table 2). Households with the lowest monthly income had the lowest awareness scores, and the score increased with increased income, although this trend was not statistically significant. The awareness score was also not statistically significant with employment status.

**Table 2:** Characteristics of participants and sociodemographic determinants associated with awareness towards hepatitis B (*n* = 764)

Variable	Frequency (%)	Mean score (±SD)	*p*-value	Awareness (good/poor)	Univariate logistic (good vs. poor)		Multivariate logistic (good vs. poor)	
					OR (95% CI)	*p*-value	OR (95% CI)	*p*-value
Gender			0.124					
Male (R)	416 (54.5)	1.89 (1.31)		151/265	1		1	
Female	348 (45.5)	2.09 (1.30)		146/202	1.27 (0.95–1.70)	0.110**	1.53 (1.07–2.18)	0.019
Age (years old)			< 0.001**					
25–34 (R)	275 (36.0)	1.70 (1.28)		76/199	1		1	
35–44	232 (30.4)	2.17 (1.20)		107/125	2.24 (1.55–3.24)	< 0.001**	2.07 (1.36–3.16)	0.001**
45–54	162 (21.2)	2.04 (1.35)		68/94	1.89 (1.26–2.85)	0.002**	1.81 (1.12–2.91)	0.015*
55 and above	95 (12.4)	2.24 (1.46)		46/49	2.46 (1.52–3.98)	< 0.001**	2.95 (1.58–5.53)	0.001**
Ethnicity			0.001**					
Indian (R)	125 (16.4)	1.86 (1.35)		43/82	1		1	
Malay	453 (59.3)	1.87 (1.27)		157/296	1.01 (0.67–1.53)	0.957	0.96 (0.62–1.49)	0.848
Chinese	182 (23.8)	2.32 (1.34)		95/87	2.08 (1.30–3.33)	0.002**	1.75 (1.06–2.89)	0.027*
Others	4 (0.5)	2.25 (1.50)		2/2	1.91 (0.26–14.01)	0.526	2.38 (0.29–19.43)	0.420
Marital status			< 0.001**					
Single (R)	147 (19.2)	1.61 (1.32)		44/103	1		1	
Married	600 (78.5)	2.08 (1.30)		248/352	1.65 (1.12–2.43)	0.012*	1.30 (0.79–2.12)	0.302
Widowed and divorced	17 (2.2)	1.53 (1.13)		5/12	0.98 (0.32–2.93)	0.965	0.88 (0.26–2.94)	0.829
Employment status			0.745					
Public sectors (R)	101 (13.2)	1.94 (1.30)		36/65	1		1	
Private sectors	222 (29.1)	1.95 (1.35)		85/137	1.12 (0.69–1.83)	0.649	1.21 (0.72–2.02)	0.480
Self-employment	184 (24.1)	2.04 (1.28)		77/107	1.30 (0.79–2.15)	0.306	1.28 (0.74–2.20)	0.382
Student	27 (3.5)	1.78 (1.22)		8/19	0.76 (0.30–1.91)	0.560	1.36 (0.49–3.75)	0.556
Retired	54 (7.1)	2.13 (1.42)		24/30	1.44 (0.74–2.83)	0.285	1.07 (0.48–2.36)	0.873
Unemployed	156 (20.4)	1.92 (1.24)		57/99	1.04 (0.62–1.75)	0.884	0.96 (0.53–1.74)	0.889
Others	20 (2.6)	2.30 (1.53)		10/10	1.81 (0.69–4.75)	0.231	1.411 (0.50–3.98)	0.516
Educational attainment			< 0.001**					
Illiterate or primary school (R)	51 (6.7)	1.27 (1.20)		10/41	1		1	
Secondary education	361 (47.3)	1.99 (1.32)		146/215	2.78 (1.35–5.73)	0.005**	3.58 (1.66–7.71)	0.001**
Diploma/ certificate	193 (25.3)	2.02 (1.24)		77/116	2.72 (1.29–5.76)	0.009**	4.57 (2.00–10.44)	< 0.001**
Degree holder	131 (17.1)	2.02 (1.37)		49/82	2.45 (1.13–5.33)	0.024*	4.13 (1.75–9.75)	0.001**
Postgraduate	28 (3.7)	2.64 (1.16)		15/13	4.73 (1.72–13.05)	0.003**	7.60 (2.55–22.68)	< 0.001**
Family income (RM)			0.287					
≤ 2000 (R)	183 (24.0)	1.80 (1.37)		68/115	1			
2001–3000	178 (23.3)	1.98 (1.20)		71/107	1.12 (0.73–1.72)	0.594		
3001–4000	125 (16.4)	2.07 (1.36)		50/75	1.13 (0.71–1.80)	0.615		
4001–5000	88 (11.5)	2.02 (1.20)		32/56	0.97 (0.57–1.64)	0.899		
> 5000	190 (24.9)	2.07 (1.36)		76/114	1.13 (0.74–1.71)	0.573		

*Significance at 0.05

**Significance at 0.01

^a^Analysis using Analysis of Variance (ANOVA)

*CI* confidence interval, *OR* odds ratio, *R* reference group, *RM* Ringgit Malaysia, *SD* standard deviation

In this study, 297 (38.8%) respondents were categorized as having good awareness. Although more than 75% of respondents knew that there was free HepB vaccination for infants in Malaysia, only 50.0% knew their HepB status, and 45% of them knew their family members’ status. Only 202 (26.4%) of the participants have had a HepB vaccination.

The univariate logistic regression analysis revealed that older age, Chinese ethnicity, marriage status, and high educational attainment were all associated with good awareness (*p* < 0.05) (Table 2). Gender, employment status, and family income were not associated with awareness of HepB. Compared to the youngest group, participants aged 35–44 years (OR: 2.24), 45–54 years (OR: 1.89), and 55 years or above (OR: 2.46) had increased odds of having good awareness. Being Chinese, but not Malay, increased the odds of having good awareness (OR: 2.08; 95% CI: 1.30–3.33) compared to being Indian. Married participants also had almost double the odds of having good awareness compared to unmarried participants. Compared to participants with a primary school or lower education, the odds of having good awareness increased for participants who finished secondary education (OR: 2.78; 95% CI: 1.35–5.73), who had completed a diploma certificate (OR: 2.72; 95%CI: 1.29–5.76), who had a bachelor’s degree (OR: 2.45; 95% CI: 1.13–5.33) or who had a postgraduate degree (OR: 4.73; 95%CI: 1.72–13.05).

The multivariate model revealed that all associated variables in the univariate model, except for marital status, were significantly associated with good awareness. Similar to the knowledge domain, high education was the strongest predictor for good awareness.

### Association between knowledge and awareness towards hepatitis B

Using Spearman’s rank correlation (r_s_), our data indicated a significant positive correlation between knowledge scores and awareness scores (r_s_: 0.246; 95% CI: 0.177–0.312, *p* < 0.001). Similarly, another analysis comparing good knowledge and good awareness showed that participants who had good knowledge were 2.5 times more likely to have a good awareness (OR: 2.41, 95% CI: 1.78–3.26, *p* < 0.001).

## Discussion

The government of Malaysia through the Ministry of Health has formulated a road map to eliminate viral hepatitis infection in the country by 2030. Prevention of both vertical and horizontal transmission is one of the key strategies to reduce the incidence of HepB. This measure can only succeed if community members have good knowledge and awareness of the infection because it requires comprehensive participation of community members. Therefore, information related to knowledge and awareness is very essential not only to understand deficiencies in knowledge and awareness in the community but also to design intervention programs. Due to limitations of existing information and in order to provide more comprehensive data, we conducted a community-based cross-sectional survey covering Selangor state, the most populous state in Malaysia, which is home to over 6.3 million Malaysians.

We found that only 36.9% of the participants had good knowledge of HepB. This finding is comparable with previous studies in Malaysia. A study of community members, healthcare workers and university students found 39.1% of respondents had good knowledge (using a cut-off point of 73.3%) [10]. Among university students (undergraduate, master and PhD students), 50.3% of the respondents had good knowledge (using a cut-off point of the median score) [8]. In a population of people with chronic HepB, the mean knowledge score was only 12.57/20 (62.85%) [7]. In this study, 38.8% of respondents are categorized as have good awareness. Low awareness towards HepB has also been reported among community members [10] and among dentists [11] in Malaysia. Together, these figures indicate that knowledge [8–10] and awareness [10, 11] towards HepB is low in Malaysia. The lack of knowledge and awareness of HepB is a major obstacle for putting forth an effective healthcare agenda, and also has implications for the continued spread of the infection. Our findings of low knowledge and awareness highlight the need to improve public knowledge and awareness towards HepB through the dissemination of information on HepB to community members in Malaysia.

One of the strongest predictors for poor knowledge and awareness towards HepB is low education. The impact of education on good knowledge of HepB has been reported in studies from Australia [34], British Columbia, Canada [35], Canada [36], China [37], Kenya [38], Malaysia [7], Poland [39], Singapore [40], and among Cambodian Americans in the US [41]. Furthermore, one study also found that education level was associated with both HBV screening and HBV vaccination [42]. There are at least two reasons for this finding. Firstly, HepB is a complex disease with variations in natural history, progression and clinical management; individuals with low levels of education could have difficulty in understanding and interpreting information related to HepB. Secondly, individuals with higher education have greater access to information related to HepB from various sources and therefore are more likely to have better knowledge. These findings have two important implications. Firstly, community members with low educational attainment are the most appropriate group to be targeted in intervention programs to improve knowledge and attitudes towards HepB in Malaysia. Secondly, information related to HepB being used in prevention programs needs to be simplified so that it is easy to understand for households with low academic education.

Another important finding from our study is the impact of ethnicity on knowledge and awareness of HepB. We found that, compared to Indians, individuals with Malay ethnicity were associated with having good knowledge while Chinese ethnicity was associated with having good awareness. The significance of these associations are robust to changes in categorization (i.e. Indian, Chinese and others for Malay; Indian, Malay and others for Chinese). One previous study found that the number of carriers of HBsAg and the number of individuals with cirrhosis are higher among Chinese Malaysians compared to other ethnicities in Malaysia [43]. HepB was the predominant etiology for cirrhosis among Chinese (58.8%), whereas it was a less dominant etiology for Malays (47.9%) and Indians (5.6%) [43]. In China, up to 70% of cirrhosis and 80% of hepatocellular carcinoma are attributable to HBV infection [44]. If these figures are similar among ethnic Chinese in Malaysia, the relatively high prevalence of HepB among Chinese Malaysians could explain their greater awareness of HepB. For example, more than 50 and 66% of them knew the status of their family members and themselves, respectively, compared to 42 and 45% among other ethnicities. Accordingly, the more someone has had experience or exposure to a condition, the better awareness they have. Interestingly, although Malaysian Chinese had higher awareness, their knowledge was lower than Malays. Altogether, these findings are important for designing HepB prevention program for several reasons. First, prevention and control programs should be designed with target ethnicities in mind and can be adjusted to reflect different cultural backgrounds. Second, programs should be designed not only to increase knowledge but also awareness – a topic critically important for the Malay ethnicity – in order to translate knowledge into real preventive practices. In addition, like in the case of Human Immunodeficiency Virus (HIV) [45], social stigma is prevalent in some communities and this has caused several complications for both the patients and the associated medical system. Social stigma can be associated with the disclosure of an individual’s HBV status [44, 46]. To anticipate consequences of stigma, the program should also work to reduce the social stigma associated with HepB.

This study had some strengths and limitations. Participants might tend to give favourable answers during the interview as a form of social desirability bias. A strength of this study is that we attempted a random selection from the population, and we therefore have confidence in the ability of our results to generalize to the population of Selangor. Nonetheless, the population may differ substantively from other countries of the world.

## Conclusion

Although HepB has been a major public health concern in Malaysia for a long time and results in more deaths than any other vaccine-preventable disease, knowledge and awareness of HepB among households in the country is relatively low. Therefore, well-designed preventive programs which not only increase knowledge and awareness but also increase HepB preventive practices among households is required. This program should be designed to target population groups with low knowledge and infection prevelance (such as Indians and those with low education). We also recommend that educational materials should seek to eliminate social stigma associated with HepB because stigma is a potentially major barrier to the successful implementation of preventive, diagnostic and treatment programs.

## Additional files

Additional file 1: **Table S1.** STROBE checklist of the study. (PDF 162 kb)

Additional file 2: **Table S2.** Questionnaire used in the study. (PDF 201 kb)

## Abbreviations

95% CI: 95% confidence intervals
ANOVA: Analysis of Variance
HBsAg: Hepatitis B virus surface antigen
HBV: Hepatitis B virus
HepB: Hepatitis B
OR: Odds ratio
rs: Spearman’`s rank correlation
