# Supplementary material for: Knowledge and awareness of hepatitis B among households in Malaysia: a community-based cross-sectional survey
Source: BMC Public Health. 2019 Jan 10;19:47. doi: 10.1186/s12889-018-6375-8 (PMC6327400; doi:10.1186/s12889-018-6375-8)
Supplement: Supplementary file 1 — Table S1. STROBE checklist of the study. (PDF 162 kb) [file 12889_2018_6375_MOESM1_ESM.pdf]

Additional file 1 - STROBE checklist of the study

Items	Item No	Recommendation	Subheading of article
**Title and abstract**	1	(a) Indicate the study's design with a commonly used term in the title or the abstract<br>*Knowledge and awareness of hepatitis B among households in Malaysia: A community-based cross-sectional survey*	*Title*
		(b) Provide in the abstract an informative and balanced summary of what was done and what was found<br>*Abstract in this study consisting of background, method, results and conclusion sections with informative and balanced information.*	*Abstract*
**Introduction**			
Background/rationale	2	Explain the scientific background and rationale for the investigation being reported<br>*We provided specific background that hepatitis B (HepB) is prevalent in Malaysian but the data related to knowledge and awareness among Malaysians is very limited. This knowledge is important for used to design intervention strategies at a national scale and to develop an effective HepB prevention program. Therefore, this study was conducted to provide more comprehensive data related to knowledge and awareness in Malaysia.*	*Background*
Objectives	3	State specific objectives, including any prespecified hypotheses (N/A)<br>*"The aim of this study was to assess the knowledge and awareness of HepB and to identify associated sociodemographic determinants among representative community members in Malaysia."*	*Background*
**Methods**			
Study design	4	Present key elements of study design early in the paper<br>*This study was cross-sectional study. "A cross-sectional survey was conducted in Selangor state which is located on the west coast of peninsular Malaysia and which encircles the capital Kuala Lumpur".*	*Methods: Study setting*
Setting	5	Describe the setting, locations, and relevant dates, including periods of recruitment (N/A), exposure (N/A), follow-up (N/A), and data collection.<br>*Setting of study: cross-sectional survey among households in Selangor state of Malaysia. Locations of study: Selangor state which is located on the west coast of peninsular Malaysia and which encircles the capital Kuala Lumpur. The state is the most populated state in Malaysia with a surface area of 8,104 km2 and a population of 5.79 million. Relevant dates of study or data collection: Between January and May 2016. Data collection was conducted: face-to-face interview was conducted in Malay or English by trained interviewers.*	*Methods: Study setting & Data collection*
Participants	6	(a) Give the eligibility criteria, and the sources and methods of selection of participants<br>*Eligible criteria in this study: One adult aged 20 years or above who was a Malaysian citizen was selected and invited to participate from selected household. Method of selection: "To capture a representative sample from the population, a two-stage cluster sampling design with proportional allocation was employed with assistance from the Malaysia Department of Statistics. Briefly, the study area was divided into 16,562 enumeration blocks consisting 80-120 living quarters and 64 enumeration blocks, and 12 living quarters from each enumeration blocks were selected randomly."*	*Methods: Sampling method*
Variables	7	Clearly define all outcomes, exposures (N/A), predictors, potential confounders, and effect modifiers (N/A). Give diagnostic criteria (N/A)<br>*Response variables or outcome of the study: The response variables in this study were knowledge and awareness of HepB among community members in Malaysia. Explanatory variables or predictors in this study: gender, age, ethnicity, marital status, employment status, educational attainment and family income level.*	*Methods: Study variables*
Data sources/ measurement	8*	For each variable of interest, give sources of data and details of methods of assessment (measurement). Describe comparability of assessment methods if there is more than one group.<br>*Interest variables (included explanatory and response variables) were assessed by interviews. The differences of the mean score of knowledge and awareness between explanatory variables were analyzed using Analysis of Variance (ANOVA) and a multi-step logistic regression analysis.*	*Methods: Data collection & Statistical analysis*
Bias	9	Describe any efforts to address potential sources of bias<br>*During interview, the correct answers to the survey questions were not provided to interviewers. In addition, confounding factors were explored between the adjusted odds ratio (aOR) in multivariate analyses and the crude odds ratio (OR) in univariate analyses using strategy that have been described previously.*	*Methods: Data collection & Statistical analysis*
Study size	10	Explain how the study size was arrived at<br>*In this study, sample size was calculated using suggestions from Mitchell and Carson. The minimum sample size required for this study was 683.*	*Methods: Sampling method*

Items	Item No	Recommendation	Subheading of article
Quantitative variables	11	Explain how quantitative variables were handled in the analyses. If applicable, describe which groupings were chosen and why<br>*All explanatory variables were divided into group to give quantitative measures. "The ethnicity of the participants was grouped into Malay, Chinese, Indian and others. Educational attainment, defined as the highest level of formal education completed, was classified into illiterate or primary school, secondary school, diploma, degree and postgraduate. The age was divided into four groups (25-34, 35-44, 45-54 and 55 years old or above). For employment status, five general types were used for classification: public sector, private sector, self-employment, student or university student and retired. Participants who had an unclassified job were grouped as others, and participants who had no job currently were listed as unemployed." Response variables (knowledge and awareness towards HepB) was assessed using a scoring system. For statistical analysis, the knowledge and awareness domains were dichotomized into "good" and "poor" based on a 75% cut-off point. These processes resulted all variables become quantitative and therefore suitable for further analyses.*	*Methods: Explanatory variables & Statistical analysis*
Statistical methods	12	(*a*) Describe all statistical methods, including those used to control for confounding<br>*"The differences of the mean score of knowledge and awareness between explanatory variables were analyzed using Analysis of Variance (ANOVA)". "To assess the association between the explanatory variables and the response variables, a multi-step logistic regression analysis was employed."*	*Methods: Statistical analysis*
		(*b*) Describe any methods used to examine subgroups and interactions<br>*In this study, "the correlation between scores of knowledge and awareness was assessed using Spearman`s rank correlation (rs) based on the Kolmogorov–Smirnov normality test."*	*Methods: Statistical analysis*
		(*c*) Explain how missing data were addressed<br>*In this study, we only included data of participants who provided or completed all section of the questionnaire. All participants with missing data were excluded from analyses.*	*Result: Socio demographic characteristics*
		(*d*) If applicable, describe analytical methods taking account of sampling strategy<br>*There is no problem related sampling strategy in our study, but the analytical analysis in this study was choose based on distribution of our data. For example, we used Spearman's rank correlation (r_s) in correlation analysis based on normality test of the data using Kolmogorov–Smirnov test.*	*Methods: Statistical analysis*
		(*e*) Describe any sensitivity analyses<br>*There is no any sensitivity analysis relevant to this study. However, we did questionnaire validity test to assess the internal consistency of the questionnaire prior used in the study.*	*Methods: Questionnaire development and testing*
**Results**			
Participants	13*	(a) Report numbers of individuals at each stage of study—eg numbers potentially eligible, examined for eligibility, confirmed eligible, included in the study, completing follow-up (N/A), and analysed.<br>*"In this study, 768 respondents were interviewed and four responses were excluded due to incomplete information leaving a dataset with a total of 764 (99.4%) participants".*	*Results: Socio demographic characteristics*
		(b) Give reasons for non-participation at each stage<br>*In this study, the non-participant occurred in one stage only which was incomplete data during data collection. All incomplete data from participants were excluded from the analysis.*	*Results: Socio demographic characteristics*
		(c) Consider use of a flow diagram	
Descriptive data	14*	(a) Give characteristics of study participants (eg demographic, clinical, social) and information on exposures and potential confounders<br>*In this study, characteristics of study participants are summarized Results: Socio demographic characteristics. We included a very little information of the Table 1 into description text to avoid repetitive.*	*Results: Socio demographic characteristics*
		(b) Indicate number of participants with missing data for each variable of interest<br>*In this study, we only included data of participants who provided or completed all section of the questionnaire. Meaning that each variable of interest had the same number of participants.*	*Results: Socio demographic characteristics*
Outcome data	15*	Report numbers of outcome events or summary measures<br>*The knowledge and awareness domains were dichotomized into "good" and "poor" based on a 75% cut-off point*	*Methods: Statistical analysis & Results*

Items	Item No	Recommendation	Subheading of article
Main results	16	(*a*) Give unadjusted estimates and, if applicable, confounder-adjusted estimates and their precision (eg, 95% confidence interval). Make clear which confounders were adjusted for and why they were included.<br>*In this study, unadjusted estimates (univariate analysis) and adjusted estimates are calculated for each explanatory and response variable and both of them provided in Table 1 and 2 for knowledge and awareness, respectively.*	*Table 1 & Table 2.*
		(*b*) Report category boundaries when continuous variables were categorized<br>*In this study, for statistical analysis, the knowledge and awareness domains were dichotomized into "good" and "poor" based on a 75% cut-off point (i.e. 17 or more and 3 or more were categorized good for knowledge and awareness, respectively). These category criteria used throughout the manuscript.*	*Methods: Statistical analysis*
		(*c*) If relevant, consider translating estimates of relative risk into absolute risk for a meaningful time period	
Other analyses	17	Report other analyses done—eg analyses of subgroups and interactions, and sensitivity analyses (N/A)	*N/A*
**Discussion**			
Key results	18	Summarise key results with reference to study objectives<br>*The key findings are explained throughout the discussion section with comparison with other studies.*	*Discussion*
Limitations	19	Discuss limitations of the study, taking into account sources of potential bias or imprecision. Discuss both direction and magnitude of any potential bias.<br>*Here we discussed the limitation of our study including "Participants might tend to give favourable answers during the interview as a form of social desirability bias"*	*Discussion*
Interpretation	20	Give a cautious overall interpretation of results considering objectives, limitations, multiplicity of analyses, results from similar studies, and other relevant evidence.<br>*Some cautious are given in the discussion related to our finding such as the precaution to generalise our finding in other regions. We wrote: "Nonetheless, the population may differ significantly from other countries of the world."*	*Discussion*
Generalisability	21	Discuss the generalisability (external validity) of the study results.<br>*Some generalisabilities of the results from this study were discussed especially in the larger context of Malaysian.*	*Discussion*
**Other information**			
Funding	22	Give the source of funding and the role of the funders for the present study and, if applicable, for the original study on which the present article is based.<br><br>*Funding: This study was funded by the Fundamental Research Grant Scheme (FRGS), grant number FRGS-5524635 and UPM Putra grant number 9520900.*	*Funding*

Note: An Explanation and Elaboration article discusses each checklist item and gives methodological background and published examples of transparent reporting. The STROBE checklist is best used in conjunction with this article (freely available on the Web sites of PLoS Medicine at http://www.plosmedicine.org/, Annals of Internal Medicine at http://www.annals.org/, and Epidemiology at http://www.epidem.com/). Information on the STROBE Initiative is available at www.strobe-statement.org.
