# Supplementary material for: Knowledge and awareness of hepatitis B among households in Malaysia: a community-based cross-sectional survey
Source: BMC Public Health. 2019 Jan 10;19:47. doi: 10.1186/s12889-018-6375-8 (PMC6327400; doi:10.1186/s12889-018-6375-8)
Supplement: Supplementary file 2 — Table S2. Questionnaire used in the study. (PDF 201 kb) [file 12889_2018_6375_MOESM2_ESM.pdf]

Additional file 2 - Questionnaire used in the study

Demographic data

1. Age : ☐ Years

2. Gender
- ☐ Male
- ☐ Female

3. Race
- ☐ Malay
- ☐ Chinese
- ☐ Indian
- ☐ Others: ______________________

4. Marital status
- ☐ Single
- ☐ Married
- ☐ Divorced
- ☐ Widow/Widower

5. Occupation
- ☐ Public sector
- ☐ Private sector
- ☐ S Self-employed
- ☐ Student
- ☐ Retired
- ☐ Unemployment
- ☐ Others: ______________________

6. Educational attainment
- ☐ Never been to school
- ☐ Primary school
- ☐ Secondary school
- ☐ Diploma/certificate
- ☐ Undergraduate
- ☐ Post graduate

7. Household monthly income: ☐ RM

Knowledge of Hepatitis B

No	Question	Yes	No
1	Hepatitis B infection is caused by a virus	☐	☐
2	The brain can be affected by Hepatitis B infection	☐	☐
3	The heart can be affected by Hepatitis B infection	☐	☐
4	Kidneys are affected by Hepatitis B infection	☐	☐
5	Hepatitis B can be transmitted through food or drink	☐	☐
6	Hepatitis B can be transmitted through blood	☐	☐
7	Hepatitis B can be transmitted through tattoos	☐	☐
8	Hepatitis B can be transmitted through sex	☐	☐
9	Hepatitis B can be transmitted through sharing needles	☐	☐
10	Hepatitis B can be spread through infected mother to infant hepatitis B during pregnancy	☐	☐
11	Hepatitis B can be spread by shaking hands with an infected person	☐	☐
12	Hepatitis B infection can be prevented with a vaccine against hepatitis B	☐	☐
13	Hepatitis B infection can be prevented with exercise	☐	☐
14	Hepatitis B infection can be prevented with a balanced diet	☐	☐
15	Hepatitis B infection can be prevented with good hand hygiene	☐	☐
16	There is a blood test to detect Hepatitis B infection	☐	☐
17	There are antiviral therapy for hepatitis B	☐	☐
18	Hepatitis B is a risk factor for liver cancer	☐	☐
19	Hepatitis B infection can be transmitted to your partner	☐	☐
20	The Malaysian Government provides free Hepatitis B vaccine to newborns	☐	☐
21	A complete set of Hepatitis B vaccine requires three injections of vaccines	☐	☐
22	Jaundice is one of the most common signs of Hepatitis B infection	☐	☐

Awarness of Hepatitis B

No	Question	Yes	No	Do not know
1	Government provides free HB vaccination for infants in Malaysia	☐	☐	☐
2	I know the status of my family members Hepatitis B	☐	☐	☐
3	I know my status Hepatitis B	☐	☐	☐
4	I have complete Hepatitis B vaccination	☐	☐	☐

Data demografis

1. Umur : ☐ Tahun

2. Jantina
   - ☐ Lelaki
   - ☐ Perempuan

3. Keturunan
   - ☐ Malayu
   - ☐ Cina
   - ☐ India
   - ☐ Lain-lain: ________________________

4. Taraf perkahwinan:
   - ☐ Bujang
   - ☐ Kahwin
   - ☐ Bercerai
   - ☐ Janda/Duda

5. Occupation
   - ☐ Sektor awam
   - ☐ Sektor swasta
   - ☐ Bekerja sendiri
   - ☐ Pelajar
   - ☐ Bersara
   - ☐ Tidak bekerja
   - ☐ Lain-lain: ________________________

6. Pendidikan
   - ☐ Tidak pernah kesekolah
   - ☐ Sekolah Rendah
   - ☐ Sekolah Menengah
   - ☐ Diploma/Sijil
   - ☐ Sarjana Muda
   - ☐ Pascasiswazah

7. Berapakah pendapatan bulanan isirumah Anda: ☐ RM

Pengetahuan terhadap Hepatitis B

No	Pertanyaan	Benar	Salah
1	Jangkitan Hepatitis B adalah disebabkan oleh virus		
2	Otak boleh terjejas akibat jangkitan Hepatitis B		
3	Hati boleh terjejas akibat jangkitan Hepatitis B		
4	Buah Pinggang boleh terjejas akibat jangkitan Hepatitis B		
5	Hepatitis B boleh berjangkit melalui makanan atau minuman		
6	Hepatitis B boleh berjangkit melalui darah		
7	Hepatitis B boleh berjangkit melalui tattoo		
8	Hepatitis B boleh berjangkit melalui hubungan seks		
9	Hepatitis B boleh berjangkit melalui perkongsian jarum suntikan		
10	Hepatitis B boleh merebak melalui ibu yang jangkiti Hepatitis B kepada bayi ketika mengandung		
11	Hepatitis B boleh disebarkan melalui berjabat tangan dengan orang yang dijangkiti		
12	Jangkitan Hepatitis B boleh dicegah dengan vaksin hepatitis B		
13	Jangkitan Hepatitis B boleh dielakkan dengan senaman		
14	Jangkitan Hepatitis B boleh dicegah dengan diet yang seimbang		
15	Jangkitan Hepatitis B boleh dielakkan dengan kebersihan tangan yang baik		
16	Terdapat pemeriksaan darah untuk mengesan jangkitan Hepatitis B		
17	Terdapat terapi antivirus untuk jangkitan Hepatitis B		
18	Hepatitis B adalah satu faktor risiko untuk kanser hati		
19	Jangkitan Hepatitis B boleh berjangkit kepada pasangan anda		
20	Kerajaan Malaysia menyediakan Hepatitis B vaksin percuma kepada bayi yang baru lahir		
21	Satu set lengkap vaksin Hepatitis B memerlukan 3 kali suntikan vaksin		
22	Jaundis adalah salah satu daripada tanda yang biasa bagi jangkitan Hepatitis B		

Awarness terhadap Hepatitis B

No	Pertanyaan	Ya	Tidak	Tidak tahu
1	Hepatitis B vaksinasi percuma untuk bayi di klinik kesihatan kerajaan	☐	☐	☐
2	Saya tahu status Hepatitis B ahli keluarga saya	☐	☐	☐
3	Saya tahu status Hepatitis B saya	☐	☐	☐
4	Saya telah lengkap vaksinasi Hepatitis B	☐	☐	☐
